# The Problem with Delaying Measles Elimination

**DOI:** 10.3390/vaccines12070813

**Published:** 2024-07-22

**Authors:** Natasha S. Crowcroft, Anna A. Minta, Shelly Bolotin, Tania Cernuschi, Archchun Ariyarajah, Sébastien Antoni, Mick N. Mulders, Anindya S. Bose, Patrick M. O’Connor

**Affiliations:** 1 Immunization, Vaccines and Biologicals, World Health Organization, 1211 Geneva, Switzerlandmuldersm@who.int (M.N.M.); bosea@who.int (A.S.B.);; 2 Dalla Lana School of Public Health, University of Toronto, Toronto, ON M5S 3H2, Canada; 3 Centre for Vaccine Preventable Diseases, University of Toronto, Toronto, ON M5S 3H2, Canada; 4 Department of Laboratory Medicine and Pathobiology, University of Toronto, Toronto, ON M5S 3H2, Canada; 5 Public Health Ontario, Toronto, ON M5G 1V2, Canada; 6 ICES, Toronto, ON M4N 3M5, Canada

**Keywords:** Measles, elimination, vaccination programmes, waning immunity, vaccine equity

## Abstract

Measles is a highly infectious disease leading to high morbidity and mortality impacting people’s lives and economies across the globe. The measles vaccine saves more lives than any other vaccine in the Essential Programme of Immunization and is also the most cost-effective vaccine, with an extremely high return on investment. This makes achieving measles elimination through vaccination a key child health intervention, particularly in low-income countries, where the overwhelming majority of measles deaths continue to occur. All countries and regions of the world have committed to achieving measles elimination, yet many have faced challenges securing political commitment at national and global levels and predictable, timely, and flexible support from global donors, and experienced setbacks during the COVID-19 pandemic. This has happened against a backdrop of stagnant measles vaccination coverage and declining enthusiasm for vertical programmes, culminating in a World Health Organization Strategic Advisory Group of Experts (WHO SAGE) review of the feasibility of measles eradication in 2019. Sustaining the elimination of measles long term is extremely difficult, and some countries have lost or nearly lost their measles elimination status in the face of ongoing importation of cases from neighbouring or closely connected countries in which elimination had been delayed. Thus, a widening equity gap in measles immunisation coverage creates challenges for all countries, not just those facing the greatest burden of measles morbidity and mortality. Delaying elimination of measles in some countries makes it cumulatively harder for all countries to succeed for three principal reasons: increased inequity in measles immunisation coverage makes outbreaks more likely to happen and to be larger; political will is very difficult to sustain; and immunity may wane to a point that transmission is re-established. New strategies are needed to support countries and regions in their vision for a world without measles, including ways to galvanise domestic, regional and global resources and ignite the political will that is essential to make the vision a reality.

## 1. Introduction

Measles elimination is an example of a truly country-led initiative. All countries have committed to measles elimination. All six World Health Organization (WHO) regions have established measles elimination goals, following the lead of the Pan American Health Organization (PAHO). However, degrees of progress, definitions and processes for verification, as well as target dates vary [1,2,3,4,5,6,7,8].

Measles elimination is defined as the absence of endemic measles virus transmission in a country, region or other defined geographic area for ≥12 months in the presence of a high-quality surveillance system that meets the targets of key performance indicators [9]. Following the model developed for polio eradication, regions have established mechanisms for verifying that countries have achieved measles elimination based on careful work by National Verification Committees (NVCs) for Measles and Rubella elimination that is reviewed by Regional Verification Commissions (RVCs). The RVCs verify measles elimination for individual countries when sufficient evidence is presented by the NVC that endemic measles transmission has been interrupted for at least three years. The key prerequisites to achieving measles elimination are high vaccination coverage with two doses of a measles vaccine, a well-performing surveillance system that can detect measles cases quickly, and a health system that can detect and respond quickly and effectively to potential outbreaks. A country with at least 95% coverage of two doses of a measles vaccine, accredited laboratories, high-quality surveillance and monitoring, and a rapid effective outbreak response mechanism will both eliminate measles and achieve vaccine equity. Gaps in the implementation of any parts of these systems can lead to children, adolescents and adults remaining unprotected and measles outbreaks occurring.

This article looks beyond the short- to medium-term implications of delays to measles elimination to examine long-term factors and risks to consider strategically. Measles elimination is primarily the task of governments supported in the country by various key institutions and civil society organizations and within regions by various partners including UNICEF and WHO, but the global level is an important component of the measles and rubella ecosystem that is driving progress towards elimination, and this article takes a global perspective. The objective of this review is to assess what impact delays will have on the likelihood of achieving and sustaining elimination in all regions. We consider this from three key perspectives in turn: the societal impact of increasing inequity, the implications of biological aspects, such as waning measles immunity; and the importance of solidarity between nations and regions.

## 2. Context

One of the seven impact goal indicators of the current global immunisation strategy (the Immunization Agenda 2030, IA2030) is that all countries achieve the endorsed disease control, elimination and eradication targets which include measles along with maternal and neonatal tetanus, rubella and polio [10]. Despite widespread support for IA2030, there is a lack of appetite among countries, donors and partners for another disease-specific eradication programme in light of the challenges and experiences in the polio eradication efforts over the past two decades. Regardless, countries and regions have progressed their elimination ambitions by establishing region-specific target dates for the elimination of measles in all regions and in four of six regions for rubella. Based on RVC reports, by the end of 2023, 43% of countries had eliminated measles and 51% had eliminated rubella [11]. Strong routine immunisation programmes have helped countries achieve elimination, with 32% of measles eliminated or verified countries reaching the target routine first dose measles vaccine coverage of at least 95% compared with 19% of endemic countries. In addition, good surveillance is a critical determinant of success, supported by the Global Measles and Rubella Laboratory Network which provides high-quality laboratory surveillance of measles and rubella in nearly every country. However, data also show that strong routine immunisation is neither necessary nor sufficient for achieving or sustaining measles elimination since 68% of the eliminated or verified countries did not achieve the 95% routine immunisation coverage target (Table 1). Nearly all countries that have eliminated measles have done so by a combination of campaigns and various levels of routine immunisation coverage, and some have relied mainly on campaigns.

Only one entire WHO region, the Americas, has so far succeeded in eliminating measles, in 2016, but unfortunately lost that status in 2019 and is now working to regain it [12]. Three regions—Europe, Southeast Asia, and the Western Pacific—have also faced challenges in some countries but made tremendous progress in others, even during the pandemic of COVID-19. The African (AFR) and Eastern Mediterranean (EMR) WHO Regions are further behind the others, but recently EMR has verified four countries as having eliminated measles, and AFR has started the process of verifying its first countries. A coordinated and concerted global and regional approach is essential to achieve regional elimination given the very high transmission rate for measles and high population mobility between neighbouring countries and to almost anywhere for some highly connected countries and communities [13,14]. Hence, lack of progress in one country or region has an impact on other countries and regions in an increasingly interconnected world. In response, regions are working closely with countries on cross-border surveillance and collaboration. Furthermore, a coordinated effort brings economies of scale, such as opportunities to direct global funding and vaccine supply where most needed in the interest of global health security and opportunities for peer learning between countries and regions.

Measles has re-emerged in several countries following verification of elimination, for reasons related to weak health systems, vaccine access or hesitancy in the face of the reintroduction of the virus from outside the borders [15,16]. Such outbreaks cause several issues. Firstly, measles outbreaks are extremely disruptive in any setting, with severe impact on the health of those infected, contributing to poverty through health care costs and the corollaries of reduced child survival and subsequent negative impact on economic development, as well as disrupting and overburdening the health care system. Secondly, measles outbreaks in countries that had previously been verified as measles eliminated may undermine relatively fragile confidence in the whole immunisation programme of a country, and thirdly, as mentioned above, such outbreaks often spread to other countries depending on how connected the country’s population is to other parts of the world and how rapidly the outbreak transmission is interrupted. On the positive side, outbreaks often garner media attention, particularly in previously verified countries, offering an important advocacy opportunity for strengthening immunisation services and outbreak response capacity as well as increasing political attention and will to safeguard previous gains. Measles cases are also an important reminder for parents and health workers of the importance of immunisation in general.

Conversely, as time passes post-verification of measles elimination, the perceived risk of measles decreases because people no longer see measles cases, and they consequently do not see a need for vaccination—a great example of the prevention paradox [17]. As a consequence, it becomes increasingly difficult to sustain high vaccine demand and coverage, and harder to sustain measles elimination.

The theme of ‘no one is safe until everyone is safe’ perfectly describes the solidarity required between nations in making progress together in achieving measles elimination, since measles is a threat everywhere. In that context, it is important to consider the implications of delays to achieving elimination from regional and global perspectives as an important motive for bringing nations together around their common goals. A lesson from polio eradication efforts has been not to leave the most challenging countries to last. The reasons are not just because of the explicit inequity but also because this means all countries must sustain elimination for even longer periods, with ongoing risk of measles importations and outbreaks—a lesson that the measles field is trying to leverage through prioritising resources to a small group of large countries that are anticipated to have the greatest difficulty achieving and sustaining measles elimination.

The Global Immunization Strategy, IA2030, includes the concept of measles as a tracer of immunisation system strengths and weaknesses. The phrase “measles as a tracer” is used to refer to the way in which measles outbreaks shine a light on immunity gaps which reflect failures of health systems to identify and reach missed children with vaccination [18]. Tackling measles immunity gaps can generate enormous gains for the whole of a country’s immunisation programme and, if leveraged intelligently, can also generate important gains for other health and social programmes. Within IA2030, the Measles Rubella Strategic Framework 2021–2030 (MRSF) was launched in 2021 with the goal of supporting all regions in achieving their measles elimination goals [19]. MRSF is supported first and foremost by regional resolutions on measles and rubella elimination [20,21]. Global leadership and coordination for implementation of the MRSF are delivered by the Measles & Rubella Partnership which comprises the American Red Cross, the Bill and Melinda Gates Foundation (BMGF), the Global Alliance for Vaccines and Immunization (Gavi), the U.S. Centers for Disease Control and Prevention (CDC), UNICEF, the UN Foundation and WHO.

Important context for the MRSF is a WHO SAGE feasibility assessment from 2019, which indicates that measles eradication was set to be very hard to achieve within proximate timescales. Given large differences between the starting points of the six WHO regions, the fact that global coverage of both the first dose of measles- vaccine had stalled for a decade, and considerable uncertainty about when or how quickly coverage might increase, predictions of when measles transmission might be interrupted were pessimistic. The report concluded that it was premature to set a global eradication target [22], even if that is the end goal. This has set the tone for the global measles ecosystem ever since, with the reluctance of donors to support elimination activities. The report recommended the elaboration of a set of pre-conditions to meet that would signal when the world could and should go big and go fast towards the goal of measles eradication. The authors emphasised the risks of delaying elimination and recommended that all countries and regions accelerate progress towards achieving and maintaining measles and rubella elimination goals. At that time measles vaccine coverage had stagnated for a decade finishing with the largest epidemic year since 1996. Since then, global coverage has fallen further due to the COVID-19 pandemic, especially in low-income countries that are also furthest away from achieving measles elimination.

### 2.1. Implications of Delays to Measles Elimination for Health Inequity, and Equity for Elimination

**Figure 1:**
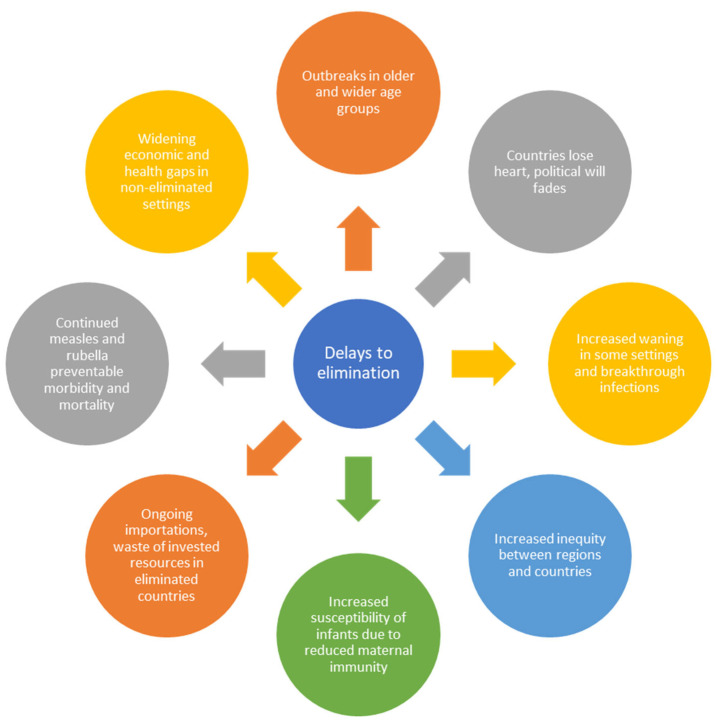
The implications of delays to achieving measles elimination.

Inequities in measles immunisation cause delays in achieving measles elimination and delays to elimination increase inequity. Delays create a vicious cycle whereby, for multiple reasons, the longer it takes a country to achieve elimination the harder it may be for that country and for other countries to succeed (Figure 1). It is therefore apt that the concept of “measles as a tracer” articulated in the Immunization Agenda 2030 incorporates the role of measles in revealing inequity in protection from vaccine-preventable diseases. The Sustainable Development Goals (SDGs) also reflect this concept in using coverage of the second dose of measles vaccine, administered during the second year of life, as an indicator of the fully vaccinated child [23].

Inequities in outcomes from measles can be conceptualised as operating at three different levels: within countries, between countries, and at the regional-global nexus. The first level gets the most focus, which is inequity within countries, for example, individuals living in rural areas have more barriers to accessing immunisation services due to weakness in primary health care, lack of government political will, lack of resources, conflict or other reasons [24]. The second level is the inequity between countries, usually assessed by some group proxy characteristic, such as gross national income. The third level is a combined regional/global nexus that relates to the will and capacity of regional and global communities to mobilise resources, catalyse progress and generate solidarity between nations.

### 2.2. Inequity within Countries

At the within-country level, inequity in protection from measles makes elimination more difficult to achieve. This is because the more unprotected individuals cluster together, the higher the coverage that is needed to interrupt transmission [25]. This occurs whatever the cause of the inequity—whether due to economic or geographical barriers, religious, racial or any other type of discrimination, or political reasons. Greater clustering of unprotected individuals also increases the likelihood and size of outbreaks. This effect has been demonstrated through mathematical modelling but is also easy to understand by imagining a thousand unvaccinated children living in one community and comparing the risk of measles with the same number of unvaccinated children spread evenly through a population of several million, in which measles transmission would be far less likely. Because measles is so infectious, any clusters of unprotected children are at risk of getting measles, and higher degrees of clustering of unprotected children increase the risk of outbreaks occurring sooner and being larger.

From an equity perspective, the children who remain unprotected from measles also tend to be disadvantaged in multiple other ways, including being at greater risk of already being malnourished as well as of becoming malnourished because of measles, and of dying from measles. Countries within the WHO African and Eastern Mediterranean Regions account for only 24% of the world’s population, yet 92% of measles deaths in 2022 occurred in these regions [9]. Measles is, therefore, not only a tracer of immunisation programme performance, but also a tracer of the outcome of that performance measured in cases and deaths, and also reflects multiple inequities in such settings (Figure 1) [26].

Within countries, excellent immunisation systems are required to achieve both equity and measles elimination since these are interdependent. We have sufficient evidence, including from increasingly diverse settings, about what it takes to succeed in achieving measles elimination. The technical tools needed to eliminate measles, which include a highly effective vaccine, systems for supply, immunisation delivery, surveillance, and data monitoring, and rapid outbreak response, are effective if fully implemented. Hence the most frequent challenges facing countries in sustaining elimination have largely been political and programmatic, including suboptimal financial and human resources investments, lack of services in hard-to-reach geographical areas [27], cold chain failures, turning children away to avoid opening a vaccine vial for one or two children (even though using one dose from a 10-dose vial is cost-effective [28]). Barriers to protection are related to failure to vaccinate rather than vaccine failure [29]. For various reasons, countries are not yet sufficiently prioritising access to primary health care. If inequities in access to measles vaccines are similar to those tracked for the third dose of diphtheria, tetanus and pertussis vaccine (DTP3) then these have been increasing since 2015 and, along with TB, account for an increasing trend in between-country inequality [30].

The reasons for low coverage and the characteristics of communities most at risk vary tremendously from place to place. In order to take appropriate action, local knowledge and high-quality local and subnational data are needed. Indeed, national data may be quite misleading. This is well-illustrated by the mismatch with measles incidence found in one country where the local incidence varied 100-fold within the country, in part due to movements of high-risk refugees due to conflict; once again, infection is shown to be concentrated in a vulnerable group [31].

In countries that have sub-optimal measles vaccination coverage, delays in achieving elimination allow the average age at infection to increase which creates multiple challenges (Figure 1). Vaccine coverage that is too low to interrupt transmission will decrease rates of infection compared to no vaccination, leaving susceptibility gaps as children age. This effect can increase the risk of measles to infants through transmission from older children and adults who had remained susceptible. Furthermore, given that nearly all countries use combined measles and rubella vaccine, this also has implications for achieving rubella elimination goals because increasing the age for rubella infection may increase congenital rubella syndrome (CRS) if susceptible children are not protected before they become adults. Measles is a tracer of rubella immunity gaps for countries using measles and rubella-containing vaccines since rubella is less infectious and may be epidemiologically silent, in contrast to measles.

Examples of measles outbreaks among older children and adults are becoming more widespread, most recently shown by outbreaks in Europe [32]. These include breakthrough infections that in general are milder and less infectious than typical measles [33,34]. Efforts to reach healthy young adults with vaccines can be more expensive and less effective than reaching children through routine immunisation system, and outbreaks in older persons are challenging to control because healthy young adults do not regularly interact with the health system and many countries lack an immunisation system for older children and adults. The increased age at time of infection can be mitigated in countries that rely on regular campaigns by widening the age range that is targeted, but funders often default to a 9–59-month age range to avoid the additional costs.

### 2.3. Inequity between Countries

With regards to inequity between countries and regions, delays and uneven progress towards achieving measles elimination have increased inequity because measles cases have become concentrated in the countries and communities facing multiple crises, including fragile economies, mass population migration, weak health systems, climate change-related disasters and conflict. These countries are more likely to have measles outbreaks and are also most likely to be disrupted by outbreaks. They are the last to benefit from measles elimination whilst having the most to benefit from elimination. Such countries are most likely to be found in the African and Eastern Mediterranean regions. Countries represented in the Americas, Europe and Western Pacific WHO regions have moved more quickly and further than other regions, leveraging political will and access to resources, and in so doing the global equity gap has widened. The Southeast Asia region has made amazing progress over the past decade, most notably in India [35].

The COVID-19 pandemic set many countries back in terms of immunisation coverage generally and measles vaccine coverage specifically, particularly in Low-Income Countries (LICs). Currently, 54% (105/194) of the world’s countries are assessed to be at high risk of experiencing measles outbreaks by the end of 2024 based on immunity profiles derived from reported routine and campaign immunisation coverage (Personal Communication Dr. Patrick O’Connor). The risk of measles outbreaks is highest in countries where coverage is lowest and where inequity within the country is very high, with LICs disproportionately experiencing both of these characteristics (Table 2). The lack of timeliness of measles vaccination campaigns in such settings is a major cause of inequity; this can and has led to thousands of preventable deaths.

### 2.4. The Global and Regional Nexus

At global, regional and country levels, the attention and commitment to measles and rubella is not commensurate with the public health burden or impact of prevention. Measles is the most cost-effective vaccine and saves more lives than any other vaccine, accounting for an estimated 60% of lives saved since the launch of EPI in 1974 [36]. Measles is one of the most cost-effective interventions a country can make, with a return on investment of more than $50 for every dollar spent [37]. Measles is also one of the diseases that display the greatest level of “negative externalities” across countries. For example, an infant in one country who is too young to be vaccinated can be infected because of a failure to control measles in another country—making it, a perfect target for multilateral engagement and resolution. Added to this, rubella is globally the most common preventable cause of congenital birth defects. Nevertheless, more energy, advocacy, and funding are dedicated to other immunisation priorities, often influenced by external interests in the use of funds within LICs. This does little to enable the establishment of strong and sustainable country-level institutions that drive equitable primary health systems, universal health care access and improved health status of populations. Such changes will not be easy to achieve and require a rebalance of power in global health which encompasses measures to enact decolonisation and raise countries’ voices [38,39].

## 3. Delays to Measles Elimination and Waning Immunity

Countries in which a large proportion of the population has become immune through measles rather than vaccination can generally rely on the life-long immunity conferred by wild measles virus infection [40]. In contrast, in eliminated settings evidence is growing that vaccine-derived immunity is not lifelong, which raises the spectre of elimination being a time-limited state in some settings and with some schedules.

Most of the available data on the waning of immunity following vaccination comes from countries that vaccinate at 12 months of age or later [41]. Even in these countries, data are somewhat limited but seem to indicate that protection wanes relatively slowly and does not fall below the threshold of protection over a timescale of many decades [42]. These estimates align well with the sustained elimination that has been achieved in many countries where routine immunisation coverage has been high enough using schedules that start earliest at 12 months of age. Emerging data on vaccination within the schedule most commonly in use in countries with endemic transmission, where the first dose of measles vaccination is usually given at 9 months, seem to indicate that waning occurs more quickly than in countries where the first dose is given at 12 months. Modelling seroprevalence data from China, where the first dose is given at 8 months of age, indicates that IgG antibody concentrations would drop below the protective threshold of 200 mIU/mL around 5 years after the first dose and 13 years after the second dose of a measles vaccine, or at 14.3 years of age [43]. These modelling findings need to be followed closely to see if they translate into reduced programmatic effectiveness. A study of the impact of an early first dose of measles vaccine in the Netherlands given at 6 months of age found much more rapid waning compared with the standard schedule starting at 14 months, resulting in more than 10% of children becoming susceptible to measles by as early as 4 years of age [44]. These data are alarming and seem to indicate the need for countries that give the first dose early to consider moving their first dose of MCV1 later to 9–12 months or potentially to introduce an additional (third) routine MCV dose. These findings also raise concern about the sustainability of measles elimination in countries that start the first dose early in a routine program.

As countries spend increasing time in a state of elimination, the likelihood increases of breakthrough infections in fully vaccinated individuals. The critical question is how transmissible breakthrough infections are. Infections in previously immune individuals are generally mild and transmit rarely to other people, and if this situation persists then they may not present a significant risk to maintaining measles elimination. Conversely, if early immunisation increases the risk of breakthrough infections and their infectiousness, then the risk arises that these may start to contribute to the epidemiology of measles transmission [45].

Based on accumulating evidence of waning immunity, delays to eliminating measles could make elimination ultimately harder to achieve as older cohorts protected by infection age out, and the immunity of younger cohorts starts waning, especially if breakthrough cases are infectious enough to jeopardise elimination [46].

A different waning immunity issue to consider affects young infants. Infants are protected during their first few months of life by maternal antibodies. Here the evidence is very clear, that mothers whose immunity is vaccine-derived pass on much lower levels of antibodies to infants compared to mothers who are immune due to previous measles infection [47,48]. This immunity is not just lower but wanes more quickly. Hence in highly vaccinated communities, infants are susceptible to measles at a much younger age than in endemic settings, and this can increase their risk should measles elimination be delayed or not sustained (Figure 1). Whether this represents a risk to infants, in general, depends largely on whether countries sustain measles elimination. In elimination settings, infants are protected by herd immunity generated by the vaccination programme until they are old enough to be protected directly by vaccination. However, they risk infection due to the importation of measles from an endemic country or through travel to such a setting.

## 4. Discussion

### 4.1. Sustainability of Elimination Is a Major Challenge and Requires Solidarity between Countries and Regions

In an ideal world, countries and regions would move forward towards elimination in coordinated lockstep because together they are stronger. As long as some regions and countries remain in a state of endemicity, all other countries have greater risk and require larger investments to maintain their elimination state. The achievement of measles elimination in the Americas illustrates this perfectly; it benefited all countries of the region by greatly reducing importations of measles due to travel within the region.

Countries that have large populations, remote communities, are federalised, have decentralised health systems, experience other health emergencies including COVID-19, climate-change-related disasters, economic crisis or conflict, waning vaccine demand and high levels of inequity, will have problems achieving and sustaining measles elimination. Several countries in these categories have achieved but then lost their elimination status; some have subsequently been reverified as having eliminated measles. The loss of measles elimination status has happened for multiple interrelated reasons including failures to address the accumulation of susceptible children, delayed campaigns, late outbreak response, mass migration, financial crisis, conflict, and vaccine hesitancy. The ideal setting for sustaining elimination is a small peaceful island state with a highly equitable society, strong universally available and accessible health care, a population that trusts in its institutions and health care providers, and with few visitors or mass movements of people. Nevertheless, the majority of the 78 countries that have achieved and sustained measles elimination have succeeded without the benefit of this idyllic setting, including countries, such as Haiti and Venezuela which have faced many challenges [49]. Such achievements are a tribute both to the countries themselves and to the community of countries and the region that have together supported each country in achieving its goal.

### 4.2. Delaying Elimination Is a Major Risk for Sustainability

Sustainability is possibly the most important determinant of success for most countries and also the hardest to achieve. It is a key attribute that has been relatively neglected in measles elimination strategies. The longer we wait, the harder it will be to sustain elimination and the more likely that measles mortality and disability will resurge. In facing challenges as enormous as measles and, in the face of constrained resources, rigorous focus is needed on what is essential.

### 4.3. Diverse Tools and Strategies Are Available to Push Forward Measles Elimination

Measles could be described as an ancient pandemic. Combat against it is ongoing. The world could learn from the COVID-19 pandemic during which equitable access to vaccines was not achieved despite attempts to coordinate globally. For measles, we have it all, with a visible disease, cheap, cost-effective and abundant vaccine and opportunities to innovate in diverse ways, such as by implementing 5-dose vials and new campaign strategies or accelerating new technology, such as MR microarray patch vaccines (MAPs) and rapid diagnostic tests (RDTs) [50,51,52]. We have the technological ability to deliver reliable accurate and timely surveillance and coverage data down to subnational levels, particularly important for understanding where action needs to be taken to raise coverage in large, decentralised and heterogeneous countries. As coverage increases in a country, the requirements for investment in high-quality information systems for surveillance and coverage increase, including home-based records, electronic immunisation registries and school entry checks to identify missed children. Where trust in vaccines has been shaken, tools and methods exist for social listening to understand vaccine confidence and how to raise it. We could build evidence on waning immunity by tracking breakthrough infections, especially in countries that start routine measles immunisation at 6, 8 or 9 months. The high-performing Global Measles Rubella Laboratory Network (GMRLN) is also a key resource for global health security and pandemic response. Recent pandemics have been respiratory diseases, as are measles and rubella. Vaccines have been key to the response and should be integral to the global health security agenda.

Such diverse tools and strategies, if implemented for measles and rubella, would not just help countries achieve and sustain measles and rubella elimination but also enhance a life course approach to immunisation and health. This is where the measles and rubella teams can work with other health initiatives to strengthen routine immunisation together, including the “second year of life platform”. School entry requirements may also help generate sustainable progress.

### 4.4. The Gap in Political Will for Measles Elimination Requires Strong Global Advocacy

We have nearly all the elements to achieve elimination but miss a key essential ingredient, which is sufficient political will to seize this opportunity. The 78 countries that have eliminated measles show us that it is possible even in very difficult settings. If momentum can be generated, many more countries are set to follow suit, but focused action is needed to ensure accelerated progress in the African and Eastern Mediterranean regions to address the regional equity gap. Lessons we can take from the Global Polio Eradication Initiative (GPEI) include the need for intensified resources in countries where the greatest impact can be made in order to raise up all countries together, relentless advocacy at all levels, strong collaboration with other initiatives, and clearly defined benchmarks and milestones towards elimination. Building momentum towards measles elimination requires not just concerted advocacy efforts but also support from global leaders in immunisation.

### 4.5. Country Voices Need to Be Elevated and Economic Benefits of Elimination Emphasised

Successful countries and regions have demonstrated that pursuing measles and rubella elimination goals relies on financing for countries as well as high-level political championing, yet resources are currently shrinking. When routine measles vaccine coverage is below 90%, countries must implement regular catch-up strategies or campaigns as a rescue strategy to reach unimmunised children and prevent outbreaks. Immunization campaigns are expensive and unpopular with country ministries of health and finance, and with donors, despite the costs of the outbreaks that occur without campaigns, but there is no escaping the need for them until routine immunisation systems are stronger. Countries with coverage below 80% face a vicious cycle of needing such frequent campaigns, often every two years, that it becomes very challenging for them to strengthen their routine immunisation systems. This campaign trap can be broken through assistance in building the strong routine immunisation systems needed to stop this rapid cycle of campaigns, but this takes time and in the interim needs to be strongly supported through a proactive and evolutionary multi-year campaign strategy. Combined with more effective and integrated health campaign planning, as is underway through the work of the Measles & Rubella Partnership (M&RP), other disease-specific initiatives and partners, more impact can be achieved with limited resources [53,54]. Helping the few countries yet to use the rubella vaccine to meet the requirements for its introduction could also be a stepping stone towards measles elimination.

We have taken a global perspective in this piece and should reflect on the implications of this viewpoint in the context of the call to decolonise global health and raise country voices [55]. Truly country-led sustainable approaches to development could force a more sustainable approach to materialise, maybe through further engagement of emerging Global South voices and leadership by large middle-income countries. In discussing inequity and measles, we should reframe the discussion from some countries being behind on progress to all countries being behind for as long as children everywhere are not being reached equally. Measles does not respect institutional boundaries, so neither should we in trying to stop its spread. There are not just health or economic benefits to be considered, there are real risks and measurable costs to failing to sustain elimination (Figure 1). For example, the question should be asked: would measles be a worthwhile investment for Development Banks established by high-burden countries? For every $1 spent, we estimate a return on investment of more than $58 [56]. There is much to be gained from investing in measles elimination.

## 5. Conclusions

We cannot expect to bring about change by repeating past strategies. New and creative solutions are desperately needed to create and drive momentum towards the agreed measles and rubella goals. At the same time, we should not delay using the strategies we know to be effective while we wait for new ones that are, as yet, untested. We already have the technical tools to finish this unfinished pandemic of measles. What is missing is political leaders and global, regional and country-level actors to join forces around a common goal and make major progress in combatting one of the world’s most burdensome diseases. Essential to these efforts is a higher ambition to address increasing inequity and strengthen health systems to reach unreached communities. The ultimate end game is to eradicate measles, but that effort cannot start until countries are ready and at the starting line for a final push, and the starting line is still far away. The resources and commitment to get there are out of step with the magnitude of the problem. It is time to correct that disparity and find the will and tenacity to act.

## Figures and Tables

**Table 1:** The strength of routine immunisation, measured by whether the first dose of measles vaccination (MCV1) coverage reaches the recommended target of at least 95% and the country’s measles elimination status.

Category	Number of Countries	MCV1 >95%	MCV1 <95%
Verified	82	28 (34%)	54 (66%)
Eliminated	21	5 (24%)	16 (76%)
Endemic	85	16 (19%)	69 (81%)
Re-established endemic transmission post-verification	5	2 (40%)	3 (60%)
No report	1	1 (100%)	0 (0%)

Data source: Regional Verification Reports shared in confidence with WHO HQ by WHO regions.

**Table 2:** Average WHO/UNICEF Estimates of National Immunization Coverage (WUENIC) of Measles-containing vaccine, 1st dose (MCV1) and Measles-containing vaccine, 2nd dose (MCV2) in 2022 and annual measles incidence per million in 2022 by Low-, Middle- and High-Income Country status (LIC, MIC and HIC).

Country Status	Dose	Coverage (%)	Incidence, Cases per Million
Low income	MCV1	66	98
Low income	MCV2	40	
Middle income	MCV1	86	18
Middle income	MCV2	79	
High income	MCV1	93	0.4
High income	MCV2	91	
Global	MCV1	83	24
Global	MCV2	74	

Source: the WHO Immunization Data Portal for coverage and cases by World Bank Income category. Available at https://immunizationdata.who.int/, accessed on 11 July 2024.
